# Supplementary material for: Novel Covariance-Based Neutrality Test of Time-Series Data Reveals Asymmetries in Ecological and Economic Systems
Source: PLoS Comput Biol. 2016 Sep 30;12(9):e1005124. doi: 10.1371/journal.pcbi.1005124 (PMC5045173; doi:10.1371/journal.pcbi.1005124)
Supplement: S1 Text — (PDF) [file pcbi.1005124.s001.pdf]

Supplemental Information

June 1, 2016

Contents

I Datasets **2**

II Constant Volatility Transformations for the Wright-Fisher Process (WFP) **3**

III Modification of KS-test to account for dependence among CVTs **5**

IV Simulation of Surrogate Data **7**

1 WFP simulation **7**

2 Parameter Estimation **8**

3 Simulating Surrogate Data for Male Tongue **9**

V PERMNOS **10**

Part I
Datasets

Microbiome datasets were obtained from

http://www.genomebiology.com/2011/12/5/R50/additional.

The collection of these data is described in Caporaso et al. 2011 [1]. All sequence count data were converted to relative abundances. The data were not collected at uniform increments, so the actual date stamps were used to calculate Δt in the data. Δt ranged from 1 to 18 days, with most Δt being 1 day.

Breeding Bird Survey data was downloaded from

https://www.pwrc.usgs.gov/bbs/rawdata/

[2] and all points along all routes in the United States were pooled, while points from Canada were ignored.

Market share and Market capitalization data were obtained by collecting share prices and share volumes from the US stock database ©2014 Center for Research in Security Prices (CRSP), The University of Chicago Booth School of Business, accessed through the Wharton Research Data Services. Out of over 500 companies in the S&P 500 from January 1, 2000 to January 1, 2005, only 459 companies were in the index continuously during that interval. One company, PERMNO 76656, recorded a negative day-end share price that appears to be a typo and was corrected - a string of day-end prices in the text file from CRSP was: 26.3200 26.6400 24.9900 -24.9300 27.7000 27.6600 27.9100. The -24.9300 was changed to 24.9300. Another company, PERMNO 10324, had a "NaN" entry in the string of share prices: 28.9100 31.5800 NaN 32.6100 35.1100; this company was removed from analysis. 6 remaining PERMNOs, 88313, 88319, 88587, 89463, 89813, 90352, and 90435, had zero entries - these PERMNOs were removed from the data. The PERMNOs for the remaining 451 S&P500 companies used in our analysis are listed at the bottom of this supplemental information file.

Share prices were converted to relative abundances to obtain market shares, and each company's capitalization (the product of share price and share volume) was converted to relative abundances to obtain the market capitalization. The dates of the day-end prices were noted and time-increments were defined as the number of days between the dates of the day-end prices (e.g. $\Delta t = 1$ for most weekdays, $\Delta t = 3$ for most weekends, etc.).

For a comparison of relative-abundance-distribution based methods of assessing neutrality, a time point was taken from each of the datasets, the counts were rarefied to 5000 for comparison across datasets (for the market capitalization, the "unit" was taken as the market share of the smallest company), and species-abundance distributions were fit using the R package 'sads'. Many of the datasets appear neutral from this perspective, and all of the datasets whose

RADs are well-fit by neutral theory's expectations are in fact rejected using our test.

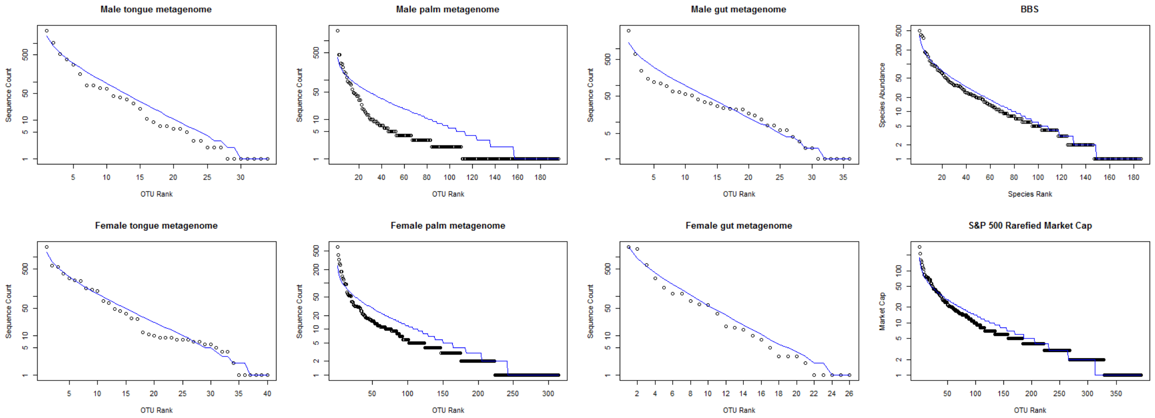

Figure 1: Empirical rank-abundance distributions (circles) plotted against the best-fit from neutral theory (blue line). Tests based on these static features may provide useful comparisons between models, but time-series data are ultimately needed to distinguish between models with similar or identical stationary properties.

Part II

Constant Volatility Transformations for the Wright-Fisher Process (WFP)

The WFP is defined by the Itô stochastic differential equation defining fluctuations in relative abundances in an n-species community, $\mathbf{X}_t = \left(X_t^1, ..., X_t^n\right)$,

$$d\mathbf{X}_t = \lambda\left(\rho - \mathbf{X}_t\right)dt + \sigma\left(\mathbf{X}_t\right)d\mathbf{W}_t \tag{1}$$

where $\lambda > 0$ and $\rho, \mathbf{X}_t \in \Delta^n$ the n-dimensional simplex, i.e. $X_t^i > 0$ for all i and t, and $X_t^1 + ... + X_t^n = 1$ for all t. The quadratic covariation between the relative abundances of different species is given by the elements of $\Sigma\left(\mathbf{X}_t\right) = {}^1\!/_2\sigma\sigma^T$, where

$$\Sigma_{i,j} = \begin{cases} X_t^i\left(1 - X_t^i\right) & i = j \\ -X_t^i X_t^j & i \neq j \end{cases} \tag{2}$$

For systems with state-dependent noise, the volatility can't be measured directly as doing so would require multiple samples from each point, allowing calculation of the covariance matrices at each point. An alternative, suitable for

testing if the volatility comes from our particular model, is to find a function, f, which has constant volatility when its input is a WFP and non-constant volatility otherwise. Thus, we seek a function $f : \Delta^n \mapsto \mathbb{R}$ such that

$$\lim_{\Delta t \to 0} \operatorname{var}\left[\frac{f(\mathbf{X}_{t+\Delta t}) - f(\mathbf{X}_t)}{\sqrt{\Delta t}}\right] = C \tag{3}$$

for some constant, C. Applying Itô's lemma to equation 1, we have that

$$\lim_{\Delta t \to 0} \operatorname{var}\left[\frac{\Delta f}{\sqrt{\Delta t}}\right] = \nabla f^T \sigma \sigma^T \nabla f. \tag{4}$$

If we let our constant be $C = 2$ a constant volatility function will be a function f such that

$$\nabla f^T \Sigma \nabla f = 1 \tag{5}$$

noting the trivial case with $C = 0$ can always be obtained by any function $f\left(X_t^1 + ... + X_t^n\right) = f(1)$. Simplifying notation by denoting $X_t^i = x_i$, equation (5) can be expanded using equation (2):

$$\sum_{i=1}^{n-1} \sum_{j>i}^{n} x_i x_j \left(\frac{\partial f}{\partial x_i} - \frac{\partial f}{\partial x_j}\right)^2 = 1. \tag{6}$$

If $n = 3$, and we denote $x_1 = x$, $x_2 = y$ and $x_3 = z$, we obtain the partial differential equation

$$xy\left(\frac{\partial f}{\partial x} - \frac{\partial f}{\partial y}\right)^2 + xz\left(\frac{\partial f}{\partial x} - \frac{\partial f}{\partial z}\right)^2 + yz\left(\frac{\partial f}{\partial y} - \frac{\partial f}{\partial z}\right)^2 = 1 \tag{7}$$

defining constant-volatility transformations for a 3-species WFP.

When $n = 2$, the PDE in equation 6 is simply

$$xy\left(\frac{\partial f}{\partial x} - \frac{\partial f}{\partial y}\right)^2 = 1 \tag{8}$$

which can be solved by substituting $u = x + y$ and $v = x - y$, simplifying to

$$\left(u^2 - v^2\right)\left(\frac{\partial f}{\partial v}\right)^2 = 1. \tag{9}$$

Since $\mathbf{X}_t \in \Delta^2$ and $u = 1$ the solution is simply

$$f = \arcsin(v). \tag{10}$$

For $n > 2$, a set of solutions can be intuited by using the grouping invariance of the WFP, i.e. that higher dimensional Wright-Fisher Processes can be collapsed into lower-dimensional Wright-Fisher processes by simply grouping species. For example, where $n = 3$, we can set $w = x + y$ and the resulting process $\mathbf{Z}_t = (w, z)$ is a 2-species WFP. Thus, $f = \arcsin(x + y - z)$ is a CVT

for $n = 3$. Since any re-grouping of species in a WFP yields a WFP, and since we always know the CVTs for the 2-species case, we can define a set of CVTs for an n-dimensional WFP:

$$f_{\mathbf{a}}(\mathbf{X}_t) = \arcsin\left(\sum_{i=1}^{n} a_i X_t^i\right) \quad (11)$$

where $a_i = \pm 1$. All 2^n of the these CVTs solve equation 6 and have constant-volatility when operating on a WFP.

Two details about this test should be noted. First, one might consider constructing a test on the magnitude - and not just the constancy - of the volatility. Doing so will provide a stronger test of the particular WFP written in equation 1, but will have a higher false-positive rate as a test of the neutrality of competition. This is clear by considering the process

$$d\mathbf{Y}_t = \lambda(\rho - \mathbf{Y}_t)\,dt + \alpha\sigma(\mathbf{Y}_t)\,d\mathbf{W}_t \quad (12)$$

formed by rescaling the volatility of a standard WFP by a scalar, α. $\mathbf{Y}_t$ has quadratic covariation $d\left[Y_t^i, Y_t^j\right] = \alpha^2 Y_t^i Y_t^j$ that is still bilinear in Y_t^i and Y_t^j and thus the species can still be grouped to form lower-dimensional WFPs - this process is still neutral in the sense that species identities do not affect their dynamics, but its volatility is a different magnitude than the WFP of equation (1). Tests for the constancy, and not the magnitude, of the volatility of $f_{\mathbf{a}}$ will provide more robust tests of the neutral symmetry - grouping invariance.

The second detail is that the 2^n constant volatility tests are redundant and some are uninformative. For instance, the sample volatilities of $f_{\mathbf{a}}$ and $f_{-\mathbf{a}}$ are equal, and the volatilities for $\mathbf{a} = (1, 1, ..., 1)$ are zero since $f_{\pm\mathbf{1}} = \arcsin(\pm 1)$. Thus, there are only $2^{n-1} - 1$informative choices of CVT.

Part III

Modification of KS-test to account for dependence among CVTs

The P-values arising from CVT tests are dependent as two CVTs may overlap greatly in their grouping of species in the community. Consequently, a standard KS-test on the distribution of P-values will have a high false-positive rate (FIGURE S1).

Correcting the high false positive rate of a standard KS-test requires accounting for the dependence among the CVTs. Chicherportiche et al. [3] developed a modified goodness of fit test for dependent data that requires calculation of copulas. However, rather than calculating copulas explicitly and using the methods developed by Chicherportiche et al., we resort to simulation of the null distribution of the KS statistic for CVTs operating on a WFP.

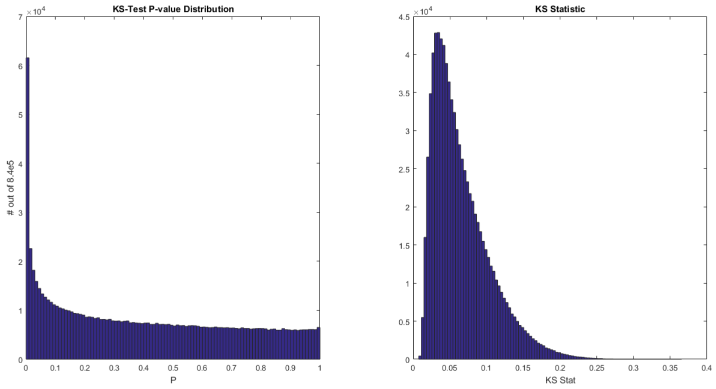

Figure 2: A standard Kolmogorov-Smirnov test on the uniformity of P-values from multiple constant-volatility tests will yield a high false-positive rate due to the dependence structure of the CVTs. **(left sub-panel)** KS tests from the 840,000 WFP simulations described here yield a high false-positive rate. **(right sub-panel)** To correct for this, we analyze distribution of KS statistics for the 840,000 simulations to parametrize the scaling of critical values of the KS statistic corresponding to $P = 0.05$ and $P = 0.005$ as functions of the number of species, n, the number of time points, T, and the number of randomly drawn CVTs, $n_{\mathbf{a}}$.

For all simulations, we used $T_{sim} = 1$ and $\lambda = 50$. The number of species, n, took 7 equally spaced values from $n = 12$ to $n = 24$. The number of time-samples, T, within the simulation window, T_{sim}, took 10 equally spaced values from $T = 125$ to $T = 1250$. The number of CVTs, $n_{\mathbf{a}}$, took values 6 in $n_{\mathbf{a}} \in \{50, 100, 200, 400, 800, 1600\}$.

Stable, numerical integration of the WFP is not trivial, especially for large numbers of species, n. The full details of the numerical integration are discussed below under "Simulation of Surrogate Data" and covered in greater detail in the thesis of Washburne (2015) [4]. For each of the 420 combinations of $\{n, T, n_{\mathbf{a}}\}$, 2,000 WFP trajectories were simulated. Using the methods from Washburne (2015), simulating the 840,000 trajectories described above resulted in only 3 simulations leaving the bounded domain, Δ^n.

We wished to understand how the $P = 0.05$ and $P = 0.005$ critical values of the KS statistic scaled with the number of species, the number of CVTs, and the number of time points. Plots of the $P = 0.05$ critical values, K_{95}, as a function of n, T, and $n_{\mathbf{a}}$are shown in figure S2. For the range of n, T, and $n_{\mathbf{a}}$ considered, K_{95} is roughly constant in T, decays as a power law for $n_{\mathbf{a}}$, and decays slower than a power law in n (figure S2). Our values of n range from $n = 335$ to $n = 1295$, well beyond the values of n simulated. For a conservative estimate of K_{95} for values of n in our analysis, we assume a power law decay in $n_{\mathbf{a}}$ and fit the critical values as a power law in $n_{\mathbf{a}}$, $K_\alpha = cn_{\mathbf{a}}^b$. We obtain $(c, b) = (0.9286, -0.4128)$ for $\alpha = 0.95$ and $(c, b) = (1.0942, -0.3968)$ for $\alpha = 0.995$.

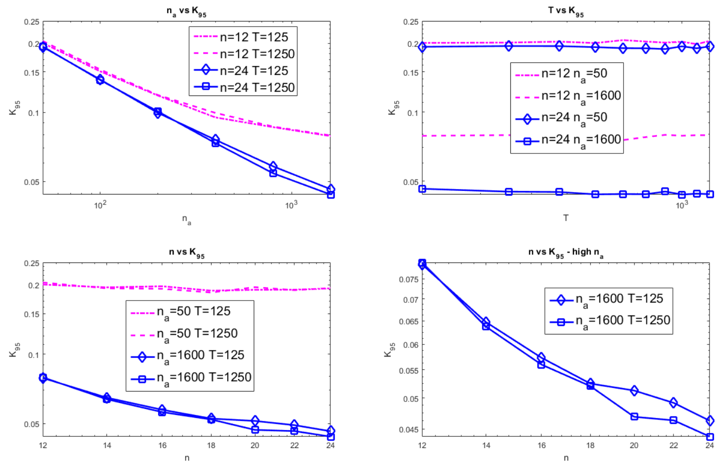

Figure 3: **(top-left)** For large numbers of species, the $P = 0.95$ critical value of the KS statistic, K_{95}, in neutral simulations follows a power law in the number of CVTs, $n_{\mathbf{a}}$. **(top-right)** K_{95} does not change significantly with T. **(bottom-left)** For large $n_{\mathbf{a}}$, K_{95} will decrease with the number of species, n. **(bottom-right)** Zooming in on the plot of n versus K_{95} for high $n_{\mathbf{a}}$. The decay in K_{95} appears to be leveling off faster than a power-law in n.

A full perturbation analysis including varying λ and T_{sim}, or an explicit calculation of the copulas, will improve the accuracy of this method for testing the neutrality of competitive systems.

Part IV
Simulation of Surrogate Data

Simulating the WFP is not trivial due to the bounded state space, Δ^n. Some tools for the numerical simulation of the WFP were developed in the thesis of Washburne (2015) [4]. The relevant tools are discussed here.

1 WFP simulation

We used Washburne's (2015) methods for simulating the WFP, namely simulating a log-ratio transformation,

$$g^i\left(\mathbf{X}_t\right) = \log\left(\frac{X_t^i}{1 - X_t^i}\right) \tag{13}$$

for $i = 1, ..., n$. Trajectories for the log-ratio transformation of the WFP were computed by integrating the SDE,

$$dg_t^i = \left(e^{g_t^i/2} + e^{-g_t^i/2}\right)^2 \left[\left(\lambda p_i - \frac{1}{2}\right) + \frac{(1-\lambda)}{1+e^{-g_t^i}} - \frac{1}{2}\left(\frac{n-2}{n}\right)\right] dt + \nabla \mathbf{g}_t^T \sigma\left(\mathbf{g}_t\right) d\mathbf{W}_t \tag{14}$$

obtained by Ito's lemma and the addition of a dimensionality correction to the drift, $-\frac{1}{2}\left(\frac{n-2}{n}\right)\left(e^{g_t^i/2} + e^{-g_t^i/2}\right)^2 dt$, shown to keep $\mathbf{X}_t \in \Delta^n$ without affecting the bilinear quadratic covariation of $\mathbf{X}_t$ [4].

We also use Washburne's method for factoring the covariance matrix, Σ, into a zero-sum matrix and a competition matrix,

$$\Sigma = PVP^T$$

where P is the matrix,

$$P = \begin{pmatrix} \mathbf{1}_{n-1}^T & \mathbf{0}_{n-2}^T & \cdots & 0 \\ & \mathbf{1}_{n-2}^T & \ddots & \vdots \\ -I_{n-1} & & \ddots & 0 \\ & -I_{n-2} & & 1 \\ & & \cdots & -1 \end{pmatrix}.$$

Here, $\mathbf{1}_k$ is the $k \times 1$ vector containing all ones, $\mathbf{0}_k$ is the $k \times 1$ vector containing all zeroes, and I_k is the $k \times k$ identity matrix. V is a diagonal matrix whose ith diagonal is the $i$the element of the vectorized, upper-triangular region of Σ. For example, if $n = 4$ and $X_t^1 = x$, $X_t^2 = y$, $X_t^3 = z$ and $X_t^4 = w$, we have the factorization

$$\Sigma = \begin{pmatrix} 1 & 1 & 1 & 0 & 0 & 0 \\ -1 & 0 & 0 & 1 & 1 & 0 \\ 0 & -1 & 0 & -1 & 0 & 1 \\ 0 & 0 & -1 & 0 & -1 & -1 \end{pmatrix} \begin{pmatrix} xy & & & & & \\ & xz & & & & \\ & & xw & & & \\ & & & yz & & \\ & & & & yw & \\ & & & & & zw \end{pmatrix} \begin{pmatrix} 1 & -1 & 0 & 0 \\ 1 & 0 & -1 & 0 \\ 1 & 0 & 0 & -1 \\ 0 & 1 & -1 & 0 \\ 0 & 1 & 0 & -1 \\ 0 & 0 & 1 & -1 \end{pmatrix}$$

.

All WFP trajectories simulated here were simulated by Euler-Maruyama integration of the dimension-corrected Itô SDE of the log-ratio transform, g, using the covariation matrix factorization described above. The trajectories of $\mathbf{g}_t$ were then mapped through the inverse of $\mathbf{g}_t$ to obtain the WFP $\mathbf{X}_t$.

2 Parameter Estimation

Surrogate datasets are constructed by numerically integrating equation (1) over a time interval, $[0, T_{sim}]$. To match at WFP to the data, we need to estimate parameters in the model, λ, ρ, and T. First, we estimate λ and ρ. Second, since

λ defines an autocorrelation time, we can use λ to estimate the timescale of the simulations, T_{sim}.

The parameters λ and ρ can be estimated by their relation to the stationary mean & variance of the WFP. Consider a two-species WFP, $n = 2$, and for simplicity let's use the notation $X_t^1 = X_t$ and $X_t^2 = Y_t$ and $\rho_1 = \rho$. Denoting the first and second moments of the WFP $m_t = E[X_t]$ and $s_t = E[(X_t)^2]$, we can use Itô's lemma and Itô's isometry to yield the system of ODEs

$$\begin{aligned} \frac{dm_t}{dt} &= \lambda(\rho - m_t) \\ \frac{ds_t}{dt} &= 2(1+\lambda\rho)m_t - 2(1+\lambda)s_t \end{aligned} \tag{15}$$

which yields the stationary mean, $m_\infty = lim_{t\to\infty} m_t$ and second moment $s_\infty = lim_{t\to\infty} s_t$

$$\begin{aligned} m_\infty &= \rho \\ s_\infty &= \frac{1+\lambda\rho}{1+\lambda} m_\infty. \end{aligned} \tag{16}$$

Thus, the stationary mean is $\mu = \rho$ and the stationary variance is given by $v^2 = s_\infty - m_\infty^2$, yielding $v^2 = \frac{\rho(1-\rho)}{1+\lambda}$. These two equations give one way of estimating λ and ρ. Given a two-species system, we estimate the sample mean, $\hat{\mu}$, and sample variance, $\hat{v}^2$, and from those obtain our estimates of λ and ρ:

$$\begin{aligned} \hat{\rho} &= \hat{\mu} \\ \hat{\lambda} &= \frac{\hat{\mu}(1-\hat{\mu})}{\hat{v}^2} - 1 \end{aligned} \tag{17}$$

The last parameter that remains to be estimated is the length of time, T_{sim}, to simulate our surrogate datasets. An estimate, $\hat{T}_{sim}$, can be obtained by noting that the true autocorrelation time of the WFP is $\tau_c = \frac{1}{\lambda}$, i.e. the autocorrelation $\mathrm{Cor}(X_t, X_{t+\tau_c}) = e^{-1}$. For a dataset with M time points, the autocorrelation function can be interpolated to find the time lag, τ with $0 \le \tau \le M$, where $C(X_t, X_{t+\tau}) = e^{-1}$. We want a surrogate dataset to be simulated for the same number of autocorrelation times as our empirical data, i.e. $\frac{\hat{T}_{sim}}{\tau_c} = \frac{M}{\tau}$, thus the time-length for the surrogate dataset will be

$$\hat{T}_{sim} = \frac{M}{\lambda\tau}. \tag{18}$$

3 Simulating Surrogate Data for Male Tongue

To produce the overlayed scatter plots of ν_t versus f_t for real & surrogate data, the male tongue was grouped into two groups: the first 200 species (as ordered in the link http://www.genomebiology.com/2011/12/5/R50/additional) formed group 1 and the remaining 173 species formed group 2. The relative abundances of group 1 was $\hat{\rho} = 0.5652$, and the rate of mean reversion estimated at

$\hat{\lambda} = 18.0063$. The estimated timescale of the dataset for simulation of the WFP was$\hat{T}_{sim} = 13.4872$. 4,000 2-species WFP trajectories were simulated by initializing the community at $X_0 = \hat{\rho}$, integrating with $\Delta t = 10^{-6}$, and sampling 332 points in $[0, \hat{T}_{sim}]$ such that the relative distance between the samples matched those of the 332 time points in the data. Of the 4,000 trajectories simulated, none left the bounded domain.

To analyze the distribution of P-values from constant-volatility tests, we generated 16,000 surrogate trajectories of f_t. To generate these surrogate data, 16,000 random groupings, $\mathbf{a}$, were chosen by randomly assigning $a_i = \pm 1$, $\hat{\rho}$ was estimated as average relative abundance of one group, $\hat{\lambda}$ was kept at $\hat{\lambda} = 18.0063$ from the previous estimation, and one WFP trajectory was simulated and subsampled as above. Generating 16,000 trajectories required discarding 11 trajectories which left the bounded domain. The mean distance from the boundary, $\delta = 0.5 - |\hat{\rho} - 0.5|$, was significantly lower in the 11 discarded trajectories compared to trajectories included in our analysis: trajectories included in our analysis had $\delta = 0.3598$, compared to $\delta = 0.0367$ for the 11 trajectories discarded. Consequently, these surrogate data had a very slight bias against extremely lop-sided groupings.

Part V

PERMNOS

10078 10104 10107 10108 10137 10138 10145 10147 10225 10401 10516 10562
10874 10890 10942 11081 11308 11404 11415 11607 11674 11703 11754 11762
11850 11896 11955 11970 11976 12052 12060 12079 12140 12431 12490 12503
12570 13100 13119 13688 13821 13856 13901 13928 14008 14277 14322 14323
14541 14593 14656 14702 14891 15069 15077 15202 15472 15560 15579 15667
15720 16424 16432 16600 16678 17005 17144 17478 17750 17806 17830 18016
18092 18163 18403 18411 18542 18729 19350 19393 19502 19561 20053 20220
20482 20626 21178 21186 21207 21371 21573 21776 21792 21936 21979 22032
22103 22111 22293 22509 22517 22592 22752 22779 22840 22947 23026 23077
23114 23229 23309 23318 23473 23579 23712 23819 23915 23931 23990 24010
24046 24109 24205 24221 24272 24360 24459 24563 24643 24766 24942 24985
25013 25081 25320 25419 25487 25769 25778 25785 25953 26112 26382 26403
26518 26710 26825 27430 27633 27713 27756 27828 27887 27959 27983 27991
28222 28484 29946 30382 30681 32379 32791 34032 34746 34817 34833 35044
35051 36397 36468 36469 37584 38156 38682 38703 39087 39490 39538 39642
39917 40061 40125 40272 40416 40539 41080 41355 41443 42024 42083 42200
42534 42796 42906 43123 43350 43449 43553 43772 44601 44644 44951 45356
45495 45671 45751 45794 46578 46674 46877 46886 47175 47466 47896 47941
48267 48274 48485 48506 48653 48725 48960 49015 49154 49373 49429 49656
49680 49905 50032 50227 50876 50906 51043 51263 51369 51377 51596 51706
52038 52337 52476 52695 52919 52978 53065 53479 53613 53831 54148 54181

54690 54827 55976 56223 56232 56274 56573 57568 57665 57817 57904 58094 58246 58318 58683 58990 59010 59176 59184 59192 59248 59328 59379 59408 59440 59459 60097 60206 60442 60506 60599 60628 60871 60943 60986 61065 61241 61399 61621 61735 62092 62148 62308 62519 62770 62834 63060 64186 64282 64311 64390 64451 64565 64936 64995 65787 65875 65883 66093 66114 66157 66181 66325 66800 67467 68144 68304 68591 69032 70092 70332 70500 70519 70536 70578 71175 71563 72726 73940 75034 75154 75175 75186 75257 75333 75489 75510 75573 75646 75789 75857 75912 76076 76090 76149 76171 76201 76226 76240 76557 76565 76605 76619 76624 76625 76638 76644 76656 76708 76712 76757 76804 76887 77063 77150 77178 77284 77418 77462 77481 77546 77605 77606 77659 77768 77976 78139 78840 78916 78927 79057 79108 79129 79212 79237 79323 79718 79879 79973 80055 80080 80266 80303 80406 80599 80913 81055 81061 81126 81138 81262 81285 81593 81655 81774 81776 82598 82643 82686 82775 83332 83421 83435 83596 83693 83699 83976 83981 84001 84020 84036 84176 84342 84372 84381 84519 85032 85073 85238 85271 85348 85522 85631 85913 85914 85926 86102 86136 86176 86305 86591 86756 86845 87055 87292 90537 90609 91380 92655

References

[1] Caporaso JG, Lauber CL, Costello EK, Berg-Lyons D, Gonzalez A, Stombaugh J, et al. Moving pictures of the human microbiome. Genome Biol. 2011;12(5):R50.

[2] Pardieck K, Ziolkowski Jr D, Hudson M. North american breeding bird survey dataset 1966-2013, version 2013.0. US Geological Survey, Patuxent Wildlife Research Center< www pwrc usgs gov/BBS/RawData. 2014;.

[3] Chicheportiche R, Bouchaud JP. Goodness-of-fit tests with dependent observations. Journal of Statistical Mechanics: Theory and Experiment. 2011;2011(09):P09003.

[4] Washburne A. Competition and Coexistence in an Unpredictable World; 2015.
